# Behind the designs: An exploration of sustainability attitudes among interior design students in Jordan

**DOI:** 10.1016/j.heliyon.2024.e36443

**Published:** 2024-08-22

**Authors:** Yaman Sokienah

**Affiliations:** Yarmouk University, Faculty of Fine Arts, Irbid, Jordan

**Keywords:** Sustainability, Interior design, Design education, Ecological responsibility, Middle East, Curriculum development, Sustainable practices

## Abstract

This research is an attempt to understand where interior design students in Jordan and the Middle East Region stand in the sustainable thinking process through education and practice and if their education on sustainability and environmental issues is enough or if it needs more development in terms of interior design programs curriculums to be more effective sustainable interior designers. The survey instrument was used to assess the attitudes of interior design students toward ecological responsibility and environmental sustainability; a modified New Ecological Paradigm Scale (NEP) was used in this research. Students enrolled in interior design programs at a Northern Jordanian university were surveyed. The sample comprised 160 interior design students from different levels of an interior design program at a public university in Jordan. In the study’s sample, the students showed more interest in the negative effect of human usage of many of the planet's resources and how this will impact the planet's ecological system. This attitude provides insight into the students’ minds regarding where they have those ideas. As design students, this can motivate them to take a proactive stand toward sustainability, which may be done through their projects in the future. However, many participants did not understand sustainable practices and strategies. This can be due to the lack of sustainability training at the university level.Based on the NEP score distribution in each section, it can be concluded that students support the concept of sustainability and reject the destruction happening to the planet. This data shows an increased awareness of environmental and ecological problems, which can positively push design students and future designers to adapt sustainable design strategies to their projects.

## 1 Introduction

Sustainability is an important and essential concept in higher education, particularly in departments that specialize in design education. For architects and interior designers, it is crucial to give priority to sustainability while designing our places and environment. Given the substantial energy and resource consumption of buildings globally, there is an immediate requirement to shift towards sustainable methods in the fields of architecture and interior design. Incorporating sustainability into the curriculum of higher education is essential to provide future designers with the necessary knowledge and abilities to tackle environmental concerns. By integrating sustainability ideas into design education, we can cultivate a mindset that appreciates the need to conserve resources and take responsibility for the environment.

Sustainability is based on using resources to meet our needs without jeopardizing the needs of future generations [1]. Sustainability concepts have been studied and practiced for a while now, and their positive results can be noticed, which proves their effectiveness [1].

In their paper [2], contributes significantly to addressing the issue of interior design students' attitudes toward environmental sustainability [2]. asked their participants to fill out a modified version of the widely used questionnaire, the New Ecological Paradigm Scale (NEP) by Ref. [1] to “Understand where students stand on environmental issues will facilitate the design of appropriate instruction to support the use of sustainable methods and products” [2].

Understanding sustainability in the context of interior design is indeed a multifaceted concept. As identified by the United Nations' Brundtland Report, sustainable development is all about meeting our present needs without hindering future generations' capacity to do the same [1]. Applying this to interior design involves more than just environmentally conscious approaches; it requires incorporating various elements, including design longevity, adaptability, wellness, and socio-cultural context, among others [3,4].

The complex task of integrating sustainability into the design process has several consequences [3]. Highlighting sustainability can spark creativity, inspiring designers to discover novel materials and methods and foster a more rounded, system-oriented design approach [5]. Nevertheless, it can also add complications to the design process. Integrating eco-friendly materials and methods could have a higher price tag or sourcing difficulties [6]. There is also a possibility of making compromises, like choosing between sustainability and aesthetic appeal or among various green objectives, adding an extra level of intricacy to design decision-making [3,7]. This underscores the importance of design education in preparing students to steer through these complexities and make enlightened design decisions that uphold sustainability principles [8].

In this study, the researcher recommended [2] research that studied Northern American students' attitude toward sustainability and applied it in Jordan as part of the Middle East. As per their recommendations, a survey of design students from a northern region university could provide additional evidence about the importance of structuring the design curriculums in a way that helps students understand the importance of sustainability and the awareness of the ecological impact of sustainable practices [2].

This research is an attempt to understand where interior design students in Jordan and the Middle East Region stand in the sustainable thinking process through education and practice and if their educations on sustainability and environmental issues are enough or if they need more development in terms of interior design programs curriculums to be more effective sustainable interior designers. Understanding students' stands on sustainability will enrich interior design programs with enough data on improving and developing environmentally aware students.

Bridging the gap and lack of understanding of sustainability in its true meaning will positively impact the results and effects of a new design project that came from the students. To understand these gaps, there need to be tangible metrics related to education and culture with the concept of sustainability to assess and improve educational curricula for the better of the world [4].

## 2 Review of the literature

The social dimension of sustainability highlights the need of developing designs that not only mitigate environmental harm but also improve the well-being of individuals and communities [2]. These considerations encompass factors such as ensuring access for everyone, promoting diversity and inclusiveness, being mindful of cultural sensitivities, and actively involving the community [2]. By incorporating social sustainability ideas into design education, we may foster the development of designers who possess both environmental awareness and social accountability [9]. Prior research has emphasized the importance of the social dimension of sustainability in the field of design education. For instance, a study conducted by Ref. [4] discovered that integrating social sustainability principles into design education can result in design solutions that are more comprehensive and sympathetic. Furthermore [[10], [11], [12], [13]], highlighted the significance of involving the community and utilizing participatory design methods to enhance social sustainability in design practice. Although the significance of social sustainability in design education is increasingly acknowledged, there are still unresolved research gaps that require attention [6]. There is a requirement for additional empirical research that investigates the influence of social sustainability principles on design results. Furthermore, further investigation is required to explore efficient teaching methods for incorporating social sustainability into design curriculum.

## 3 The concept of sustainability

In interior design, sustainability involves considering environmental factors such as using natural resources responsibly, minimizing waste, and promoting energy efficiency [3]. However, it is essential to recognize that sustainability covers broader dimensions, including social and economic aspects [[1], [3]]. Social sustainability in interior design promotes well-being, supports community and cultural identity, and strives for equality [[4], [11]]. Also, encouraging universities to offer courses in sustainability will equip students with the knowledge needed to create positive change and promote sustainable practices [2]. Moreover, by recognizing sustainability's wider dimensions, including its social and economic aspects, interior design professionals can create spaces that not only minimize environmental impact but also contribute to the well-being and prosperity of their communities.

The discourse surrounding the integration of sustainability within the field of design education has been steadily increasing in academic circles [5,14]. Notably [3], pinpointed the necessity for integrating sustainable design principles in interior architecture education, drawing attention to the pivotal role of design educators in shaping future practitioners' attitudes and approaches toward sustainability. This study presents a compelling argument for rethinking the design of education curricula to better equip students with the knowledge, attitudes, and skills required to tackle the sustainability challenges of the 21st century.

Similarly [14], work underscored the essential role of design education in promoting environmental awareness among students. This research provides important insights into how design pedagogy can foster a greater appreciation and understanding of environmental issues, facilitating the transition towards more sustainable design practices. Both studies present compelling evidence underscoring the urgency and importance of infusing sustainability into design curriculums.

When examined from a broader perspective, sustainability within higher education has also received considerable attention [15]. conducted an exhaustive review on higher education for sustainable development, delving into the challenges and opportunities posed by incorporating sustainability into university curriculums. Moreover [12], charted prospective routes toward achieving sustainability in higher education, underlining the transformative power of education as an instrument for steering societal change toward a more sustainable future.

### 3.1 Sustainability in design education

As sustainability, environmental awareness, and practice are trending, its education is embedded into the curriculum. It was found that embedding sustainable and environmental education will positively affect students' attitudes toward practicing it in their work through transferring its knowledge and values [6,16].

Furthermore, it is encouraged and recommended to create sustainable and environmentally specialized educational degrees in universities and colleges, emphasizing the importance of this issue in this era [5].

Sustainability has become increasingly important in higher education, particularly in design disciplines such as architecture and interior design. The built environment, which includes both indoor spaces and structures, has a significant impact on both the environment and the use of resources. Hence, it is imperative to prioritize sustainability in design education to equip future designers with the necessary expertise and skills to create designs that are environmentally conscious and socially responsible [4,7].

The introduction of sustainable practices in higher education is crucial for several reasons. It equips students to face the environmental difficulties that our planet is currently facing. By integrating sustainability into design education, students can acquire the information and skills needed to create designs that minimize resource use, decrease pollution, and alleviate environmental effect [8,15].

Moreover, the extent of sustainability in design education goes beyond environmental issues. Moreover, it integrates socioeconomic factors, giving great importance to the creation of designs that enhance the quality of life for both individuals and communities [17,10]. The design education curriculum and pedagogy demonstrate a complete approach to sustainability by focusing on instilling in students the ideas of social responsibility, economic feasibility, and environmental stewardship [4,11,12].

There is a strong connection between the development of personal identity in architecture and interior architecture students and the concept of sustainability [13]. Incorporating sustainability ideas into design curricula can create a strong sense of societal and environmental responsibility in students, ultimately shaping designers who are socially and environmentally conscious [4].

To promote the progress of sustainable design practices, it is essential to understand the viewpoints of designers, including architects, interior architects, industrial designers, and urban planners, in relation to sustainability. Designers' attitudes can impact the implementation of sustainable design concepts in their work and their contribution to the greater sustainability conversation within the design community. An examination of designers' viewpoints on sustainability can yield significant insights into the diverse ways in which sustainability is understood within the realm of interior design and other design disciplines [18].

It is critical to note that many students will positively perceive sustainability and consider it an improvement in their projects. However [19], found that students' perception of sustainability does not always reflect their fundamental understanding of sustainability as a practice, creating a gap in their education and implementing this in their projects.

[8] argue that providing students with the right educational experience and exercises will improve their attitude and perception of sustainability. Therefore, understanding where the students stand in terms of their attitudes toward sustainability will help in assessing the gaps in their knowledge, and this, in its turn, will create a more sensible way for the educational systems to know where to start to improve their curriculum, especially architecture and interior design programs [15].

In their research [2], found that the results were that the responses as a group were moderately pro-environmental. However, there was a noticeable part of the answers marked as undecided, and this might be due to the belief of the students who are coming from a technology boom generation to believe that technological advancements will overcome the damages caused to our planet, which enhanced their beliefs that the environment is indestructible [2].

On the other hand [2], argues that their research results indicate a weakness in the student's awareness of sustainability and ecology issues due to their education and experience. Therefore, it is suggested that higher education institutions should reconsider adding more courses on sustainability and develop their curriculums to increase the awareness of sustainability and environmental issues in a practical manner that will help the students apply this knowledge into practice [2,12].

### 3.2 Sustainability challenges

Sustainability has been studied for a long time worldwide [19,15]. However, sustainability is still facing many challenges: climate change, loss of biodiversity, and educational experiences for students [8].

Developing countries encounter significant challenges when incorporating sustainable design into their development plans [16,20]. One primary obstacle is the limited availability of comprehensive courses on sustainability offered by universities [21,22]. This lack of educational resources hinders students' ability to gain in-depth insights and practical skills in sustainable practices [14]. This issue becomes even more apparent when comparing different cultures, like the United States and developing countries. In the United States, sustainable design is on the rise, while it remains relatively underdeveloped in developing nations [[10], [11], [12], [13]].

The sustainability challenges need to be addressed by educational programs as soon as possible. Also, they must be reflected in any design program's curriculum development plans. Keeping students updated with the proper understanding and background of sustainability as a concept and its application is critical to preparing them to face the challenges as soon as they enter the practice world [7].

### 3.3 Need of the study

While sustainability has increasingly become a focal point in various sectors [15], one area that has not been fully explored is the formation and development of sustainability attitudes among interior design students [2]. As budding professionals who can significantly influence sustainable practices within the design industry [3], these students' attitudes toward sustainability can shape their future actions and overall sustainability progression within the design field.

However, current educational curriculums may not sufficiently cater to this aspect of their professional growth. An apparent disconnect seems to exist between the knowledge and application of sustainable practices among design students, pointing to the possible inadequacies of the educational strategies employed [14].

Furthermore, the interplay of various influencing factors on sustainability attitudes among interior design students is yet to be investigated comprehensively. For instance, cultural background, social factors, and media exposure in shaping these attitudes remain an under-researched area [23]. Understanding these dynamics can provide critical insights into effective strategies to foster a stronger commitment to sustainability among these students.

This research seeks to goes into these issues by examining the attitudes of interior design students toward sustainability. The study further aims to identify the factors influencing these attitudes and the nuances involved in their formation and development. Through this exploration, it is hoped that the findings can provide a more nuanced understanding of the dynamics at play, inform the creation of more effective sustainability education strategies [17], and contribute to the propagation of sustainable practices in the interior design industry.

## 4 Research methods

The research design implemented in this study is a cross-sectional survey design, leveraging the use of the New Ecological Paradigm (NEP) Scale. This design was chosen for a few reasons.

Firstly, the cross-sectional design allows for data collection from a large sample at a single point in time, making it efficient and cost-effective. This is important for a study of this nature, which aims to gather data from a broad cross-section of interior design students.

The use of the NEP Scale is central to the research design. Conceptually, this attitudinal scale measures the ecological worldviews of individuals. It captures the multidimensional nature of environmental attitudes, encapsulating beliefs about human over-reliance on natural resources, recognizing the possibility of an ecological crisis, and the need for immediate action [9,20]. Therefore, using this scale, the study is positioned to assess the sustainability consciousness of interior design students – a vital attribute for future practitioners grappling with environmental and sustainability challenges.

The decision to use the NEP Scale with the participants stems from its prior successful implementation in various cultural and disciplinary contexts. For instance Ref. [17], employed the scale in a Greek context, while [18] utilized it within an African setting. Additionally [2], demonstrated the NEP Scale's applicability within a design education context, lending credence to its suitability for this study.

Therefore, this study's research design, incorporating a cross-sectional survey using the NEP Scale, allows for an effective exploration of interior design students' sustainability consciousness. It capitalizes on the strengths of the NEP Scale in measuring ecological attitudes and mirrors successful methodological precedents in sustainability and design education research.

### 4.1 Higher and design education in Jordan

Higher education in Jordan has a significant impact on shaping the country's future by providing students with the necessary knowledge and abilities to make meaningful contributions to society. The Ministry of Higher Education and Scientific Research exercises oversight over Jordan's higher education system, ensuring uniform quality and standards throughout institutions [24]. Jordan boasts a significantly high literacy rate, and the government places great importance on education, highlighting its crucial role in driving national development.

Jordanian higher education institutions offer a wide array of programs, encompassing bachelor's, master's, and doctoral degrees in fields such as science, engineering, humanities, and social sciences [24]. The government's focus on higher education is evident in its programs that promote research and innovation, aiming to enhance the country's competitiveness and stimulate economic growth [25].

Yarmouk University, located in Irbid, Jordan, it is recognized for its commitment to academic excellence and research. Founded in 1976, this university has grown significantly and is currently regarded as one of the best educational institutions in the country. It offers a wide range of undergraduate and graduate programs in fields including humanities, sciences, engineering, and social sciences. The university is known for its vibrant campus culture, diverse student body, and emphasis on creating a stimulating educational environment. It actively participates in research programs and collaborations, so significantly contributing to Jordan's academic and cultural environment. Yarmouk University's commitment to innovation and advancement ensures that its graduates are well-prepared to meet the demands of the modern world [26].

Yarmouk University, located in Irbid, Jordan, offers a prestigious interior design program that is renowned for its comprehensive curriculum and focus on hands-on skills since its establishment in 1982 [26]. The curriculum aims to provide students with a robust grounding in design theory, technical proficiency, and hands-on practice, preparing them for prosperous careers in the field of interior design. The curriculum of Yarmouk University's interior design program combines theoretical knowledge with practical experience, providing students with the opportunity to cultivate their creativity and design abilities in a nurturing and cooperative environment. The curriculum includes a thorough semester-long course that teaches design students the principles and methodologies of sustainability. However, the curriculum need consistent development and updates to keep up with the developments in the sustainability sector.

## 5 Participants

Students enrolled in interior design programs at a Northern Jordanian university were surveyed. The sample comprised 160 interior design students from different levels of an interior design program at a public university in Jordan. The male and female ratio was close to each other, where females accumulated a %50.6, and males % 49.4 and the age range is between 18 and 40. The respondents' level was as follows: % 5.6 freshmen, %11.3 sophomores, %32.5 juniors, %33.8 seniors, and %16.9 recently graduated.

### 5.1 The NEP scale instrument

In this regard, the NEP Scale, originally formulated by Ref. [1], is valuable for measuring attitudes toward ecology or sustainability awareness.

This instrument, extensively used and validated in numerous studies across diverse cultural contexts, provides a robust measure for assessing individuals' perspectives toward environmental issues. Its application ranges from Ref. [17] study within a Greek context to Ref. [18] research in an African setting, illustrating its global relevance and applicability.

Regarding its use within design education, however, literature remains scarce [2]. offered one of the few instances where the NEP Scale was used to investigate interior design students' attitudes toward sustainability. Their study elucidated how the NEP Scale could serve as a valuable tool for uncovering how interior design students perceive and value ecological matters, thereby establishing a theoretical base for utilizing the NEP Scale in this study.

Therefore, these different strands of research play a crucial role in shaping the context and direction of this study. Building upon these foundational insights, this research aims to explore how the sustainability consciousness of interior design students, as measured by the NEP Scale, evolves in response to their exposure to sustainability concepts within their curriculum. This endeavor promises to contribute significantly to understanding how design education can foster sustainable consciousness among future practitioners.

The survey instrument utilized in this study was chosen due to its relevance to the research aims and its known reliability in assessing attitudes towards ecological responsibility and environmental sustainability. The questionnaire was adapted from The New Ecological Paradigm (NEP) Scale, a commonly utilized tool in previous studies for evaluating environmental views. By utilizing the NEP Scale, we customized the survey content to suit the particular circumstances of interior design education in Jordan, enabling us to precisely assess students' level of awareness and concern for sustainability.

The survey instrument was used to assess the attitudes of interior design students toward ecological responsibility and environmental sustainability, which was modified by Ref. [2]. The survey used in Ref. [2] research was modified from The New Ecological Paradigm Scale (NEP) developed by Ref. [9] to fit the purpose of the research.

The survey was broken down into three main sections: demographic information, knowledge of sustainability, and understanding of ecology. The first part asked folks about their age, where they were in their program, and their gender. The next two sections, focusing on sustainability and ecology, used a 5-point Likert scale with 25 items each. These questions were designed to understand how well people could develop sustainable solutions and how they saw sustainability issues. Just peek at the tables in the results section for a full rundown of the questions and items we used in the survey.

Despite the NEP survey's reputation as a reliable measuring instrument, we sought to verify its reliability for our study. Consequently, we computed the Cronbach's alpha coefficients, yielding a value of .753, which suggests a moderate to high degree of reliability. This study provides us with assurance on the coherence of the survey findings (see Table 1).

**Table 1:** Reliability test.

Cronbach's Alpha	Cronbach's Alpha Based on Standardized Items	N of Items
**.754**	.791	25

### 5.2 Sampling procedure

Using a stratified sampling technique, the study sought to correctly reflect interior design students at a university in Northern Jordan. All grades (freshman, sophomore, junior, senior, and graduate) were represented in the sample. A stratified random sampling method was employed to guarantee fair representation. Because of the large population, high degree of confidence, and a large margin of error, a sample size of 160 was chosen. A clear and strict sampling technique was used to ensure the sample was representative of the university's interior design students. The study's findings highlight the significance of this methodology for achieving generalizable results: 33.8% of the respondents were seniors, and 16.9% were recent graduates.

## 6 Data collection

In this study, the researcher collected data through an online survey shared through Google Forms. The participants were selected from a Yarmouk University Interior Design department in Northern Jordan. The design department at Yarmouk University is one of the largest design departments in the region. Instructions about the survey were given to the participating students beforehand, explaining the study's goals and the specific instructions about filling out the survey—the research goal and what was required of them. The researcher ensured anonymity and privacy were maintained by structuring and collecting the responses to the survey without any identification details about the participants, such as names or email addresses.

### 6.1 Data analysis

The Statistical Package for Social Sciences (SPSS) software was used for data analysis. Descriptive statistics and standard deviations were used explicitly to summarize the data and establish significant tendencies. Furthermore, an Analysis of Variance (ANOVA) was conducted to investigate variations in attitudes toward sustainability among student groups.

## 7 Results

This section unfolds the results of the data analysis derived from the survey responses. It is divided into three segments, corresponding to each survey part. The themes of ecology and sustainability knowledge are distinguished by their focal points and scopes.

Ecology knowledge refers to the participants' grasp of ecological matters, their perceptions of our planet's health, and their cognizance of the impacts of human activities on the environment. It encompasses ideas such as the delicate balance of nature, the exploitation of the world by humanity, and the limited resources that Earth possesses. The issue of ecological knowledge largely encompasses participants' attitudes and views on the ecological aspects of sustainability.

Conversely, sustainability knowledge relates to the participants' comprehension of sustainable design practices and their perception of how sustainability can be integrated into their design projects. It includes understanding sustainable techniques, products, building codes, and the role green organizations or certifications play in addressing environmental challenges. The theme of sustainability knowledge hones in on the participants' awareness and understanding of sustainability principles and their practical application in interior design.

## 8 Ecology knowledge section

**Fig. 1:**
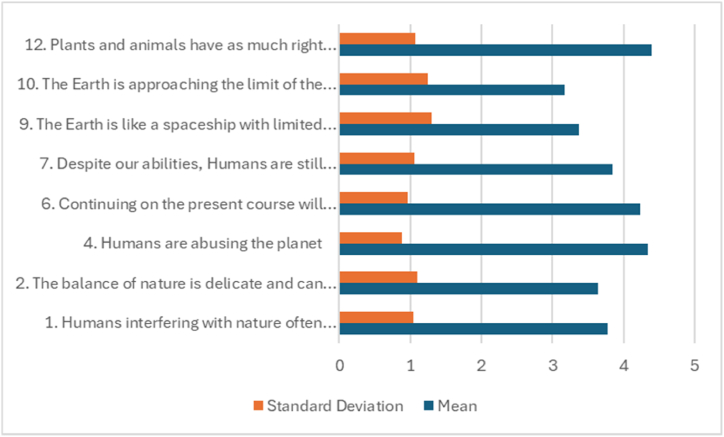
Means, and standard deviation for NEP Ecology items—positive focus.

Table 2 and Fig. 1 present participant responses to the statements from the New Ecological Paradigm (NEP) scale with a positive ecological perspective. The participants showed a recognition of humans' impacts on the environment, an appreciation for nature's delicate equilibrium, and a reverence for the existence rights of non-human entities.Item 12, "Plants and animals have as much right as humans to exist,” scored the highest mean (M = 4.40), indicating an agreement among participants. Furthermore, this result highlights an endorsement of a non-anthropocentric worldview. This showed a sentiment of biocentric equality permeating among the participants.

**Table 2:** Percentage distributions, means, and standard deviation for NEP Ecology items—positive focus (N = 160).

Statement	SD	D	U	A	SA	Mean	SD
1. Humans interfering with nature often produce unwanted consequences	3.1	10.0	19.4	41.9	25.6	**3.77**	1.041
2. The balance of nature is delicate and can be upset	3.8	13.1	22.5	36.3	24.4	**3.64**	1.101
4. Humans are abusing the planet	1.3	3.8	9.4	31.3	54.4	**4.34**	.889
6. Continuing on the present course will soon result in a significant ecological catastrophe	1.3	5.6	12.5	30.0	50.6	**4.23**	.960
7. Despite our abilities, Humans are still subject to the laws of nature	3.1	8.1	22.5	34.4	31.9	**3.84**	1.063
9. The Earth is like a spaceship with limited room and resources	10	18.8	19.4	28.1	23.8	**3.37**	1.301
10. The Earth is approaching the limit of the number of people that can be supported	11.9	17.5	29.4	24.4	16.9	**3.17**	1.245
12. Plants and animals have as much right as humans to exist	1.9	8.8	7.5	11.3	70.6	**4.40**	1.071

SD: Strongly disagree; D: disagree; U: undecided, A: Agree. SA: Strongly Agree. SD: Standard Deviation.

Of particular note, the statement “Plants and animals have as much right as humans to exist” (item 12) produced the highest mean score (M = 4.40), indicating a resounding consensus among participants. This result underscores a robust endorsement of a non-anthropocentric worldview, hinting at a sentiment of biocentric equality permeating among the student participants.

The second-highest mean score was recorded for the statement “Humans are abusing the planet” (item 4, M = 4.34). This data point underscores the participants' admission of the significant role humans play in the ongoing degradation of the planet, reinforcing the perception of an impending ecological crisis.

However, a divergence from these pro-environmental views was evident in the responses to the items focusing on the Earth's capacity to sustain human life and resource consumption. Specifically, the statements “The Earth is like a spaceship with limited room and resources” (item 9) and “The Earth is approaching the limit of the number of people that can be supported” (item 10) received notably lower mean scores (M = 3.37 and M = 3.17, respectively). This indicates a lower level of agreement with the concept of the Earth's resources and carrying capacity being finite, exposing potential gaps in participants' understanding or acceptance of these ecological concepts.

The variability in responses, indicated by the range in percentages for undecided responses (7.5 %–29.4 %) and the standard deviations, suggests diverse participant attitudes and opinions. Nevertheless, the low percentages of disagreement and strong disagreement across all statements infer a basic alignment with the positive focus of the ecological statements in the survey.

Collectively, these results imply that while the participants have a strong pro-environmental attitude, there is less consensus or understanding regarding the limitations of Earth's resources. These findings provide valuable insights for developing educational initiatives, informing future research efforts, and formulating environmental policies to address these gaps in understanding.

**Fig. 2:**
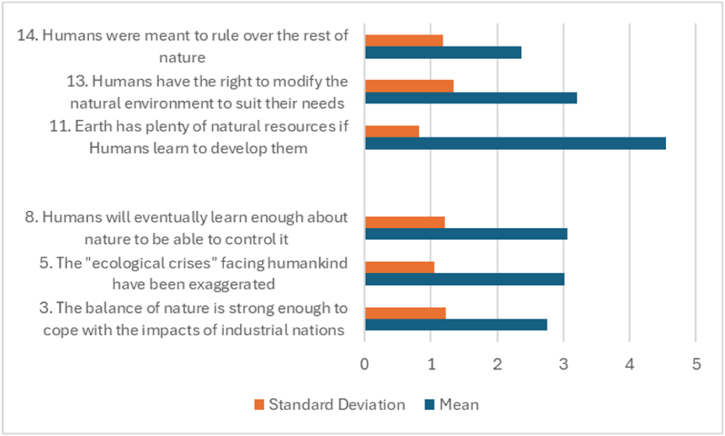
Means, and standard deviation for NEP Sustainability items—Negative focus.

Table 3 and Fig. 2 offer an insightful glance into how the participants, interior design students, perceive sustainability-related beliefs, particularly those of a negative connotation. The values provided, including percentage distributions, means, and standard deviations, create a compelling picture of their attitudes and understanding of various elements of environmental sustainability.

**Table 3:** Percentage distributions, means, and standard deviation for NEP Sustainability items—Negative focus (N = 160).

Statement	SD	D	U	A	SA	Mean	SD
3. The balance of nature is strong enough to cope with the impacts of industrial nations	11.3	43.1	16.9	15.6	13.1	**2.76**	1.231
5. The “ecological crises” facing humankind have been exaggerated	12.5	11.3	45	25	6.3	**3.01**	1.058
8. Humans will eventually learn enough about nature to be able to control it	13.1	19.4	26.3	30.6	10.6	**3.06**	1.206
11. Earth has plenty of natural resources if Humans learn to develop them	1.9	1.9	4.4	23.1	68.8	**4.55**	.823
13. Humans have the right to modify the natural environment to suit their needs	14.4	16.9	23.1	25	20.6	**3.21**	1.337
14. Humans were meant to rule over the rest of nature	29.4	30	19.4	17.5	3.8	**2.36**	1.184

SD: strongly disagree; D: disagree; U: undecided, A: Agree. SA: Strongly Agree. SD: Standard Deviation.

Among the variety of statements presented, the highest mean score is assigned to statement 11, which asserts, “Earth has plenty of natural resources if Humans learn to develop them.” The mean score of 4.55 here, accompanied by a lower standard deviation of .823, indicates a strong collective agreement among the participants. It suggests an optimism that permeates their perception of Earth's resources. It is as if they envision a world where resources are not as scarce as one might fear if humans learn to develop and manage them responsibly. This perspective resonates with the balance of nature dimension of the NEP scale, alluding to the inherent resilience of nature and its ability to recover, given humans' respectful and sustainable practices.

Similarly, statement 13, “Humans have the right to modify the natural environment to suit their needs,” achieved a high mean score of 3.21. Here, there seems to be a consensus among the participants that humans have a certain level of authority over their natural surroundings. This perspective could potentially be linked to their profession as future interior designers, where modifying spaces to meet human needs is a fundamental aspect of their work.

However, it is intriguing that the lowest mean score of 2.36 is associated with statement 14, "Humans were meant to rule over the rest of nature." This statement, reflecting an anthropocentric worldview, found minimal resonance among the students, indicating their disagreement with the idea of human domination over nature. Instead, the findings hint at a more balanced or ecological perspective among participants. This shows a promising inclination among young designers toward valuing ecological harmony and resisting an exploitative attitude towards nature, which is a refreshing observation.

When we turn our attention to statement 5, “The ‘ecological crises' facing humankind has been exaggerated,” the high percentage of undecided responses is striking. With 45 % of the respondents sitting on the fence, it hints at a certain level of uncertainty or a spectrum of opinions about the magnitude of our environmental crises. This finding is critical, as it points towards a need for further discourse and education on the subject and underlines the significance of enhancing awareness regarding the urgency and severity of environmental crises.

The standard deviations attached to each statement also offer useful insights. They hint at the range of responses and the extent of agreement or disagreement among the participants. Lower standard deviations suggest a unanimous response, while higher values indicate a broader range of opinions.

In the grand scheme of the study, these findings shed light on future interior designers' complex and nuanced perspectives. While there is an encouraging inclination towards resource conservation and an ecological perspective, the ambivalence towards the severity of ecological crises points towards gaps in awareness that need addressing.

This data underscores the importance of integrating comprehensive sustainability education into interior design curricula. Through such academic engagement, we can hope to foster a well-rounded understanding of sustainability and cultivate an ethos of respect towards the environment instead of domination. By engaging students in thoughtful discussions, educators can encourage critical thinking, empower students, and equip them to approach their future professional practice from a place of knowledge, respect, and environmental consciousness.

### 8.1 Sustainability knowledge section

**Fig. 3:**
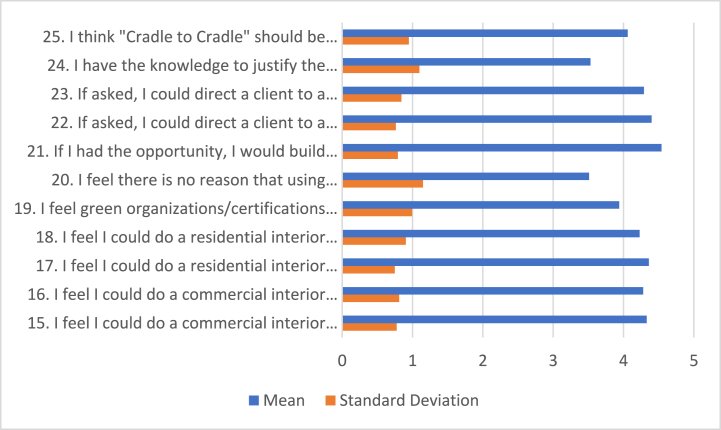
Means, and standard deviation for NEP Sustainability Statements.

Table 4 and Fig. 3 of this research presents the data associated with the sustainability-specific portion of the survey. Participants' reactions to this section were positive, implying a high level of agreement with sustainable design practices.

**Table 4:** Percentage distributions, means, and standard deviation for NEP Sustainability Statements (N = 160).

Statement	SD	D	U	A	SA	Mean	SD
15. I feel I could do a commercial interior design project using sustainable methods	0	3.1	9.4	38.8	48.8	**4.33**	.775
16. I feel I could do a commercial interior design project using sustainable products	.6	2.5	11.3	39.4	46.3	**4.28**	.810
17. I feel I could do a residential interior design project using sustainable methods	.6	1.3	8.8	40	49.4	**4.36**	.748
18. I feel I could do a residential interior design project using sustainable products	2.5	2.5	9.4	40.6	45	**4.23**	.906
19. I feel green organizations/certifications will solve environmental issues in design	2.5	4.8	26.3	32.5	35	**3.94**	.995
20. I feel there is no reason that using sustainable practices in design is optional	5	14.4	28.7	28.1	23.8	**3.51**	1.149
21. If I had the opportunity, I would build my home using sustainable methods	.6	2.5	7.5	21.3	68.1	**4.54**	.792
22. If asked, I could direct a client to a sustainable building in residential design	.6	1.9	7.5	36.9	53.1	**4.4**	.762
23. If asked, I could direct a client to a sustainable building in commercial design	1.3	1.3	13.8	35	48.8	**4.29**	.842
24. I have the knowledge to justify the added cost of sustainable features to clients	6.3	8.8	31.3	33.8	20	**3.53**	1.099
25. I think “Cradle to Cradle” should be required in an interior design program	1.9	2.5	23.1	33.1	39.4	**4.06**	.947

SD: Strongly Disagree; D: Disagree; U: Undecided, A: Agree, SA: Strongly Agree, SD: Standard Deviation.

Analyzing the mean scores for each statement, we see that most of them lean towards agreement or strong agreement. This indicates that the participants support applying sustainable practices within their design projects. However, it is important to note that items 19, 20, 24, and 25 have garnered many “undecided” responses, highlighting some uncertainty or lack of information among the students.

The item that stands out most is statement 19, which has an undecided rate of 26.3 %. This level of uncertainty is notably higher than any other statement. Statements 20, 24, and 25 also show a high proportion of undecided responses. These statements are intricately linked with the student's understanding of sustainability and ability to apply it in their work practically.

These results hint towards a deeper issue that students are grappling with. While they are positive about implementing sustainable practices and prioritizing green production in their design projects, there is a clear uncertainty regarding sustainable organizations and the building codes associated with sustainable practices.

Specifically, items 19 and 20, which examine the students' perceptions of green organizations/certifications and the necessity of sustainable practices, exhibit a wavering belief in these aspects. This could be due to a lack of familiarity or skepticism regarding the effectiveness of such organizations or the practicality of strict adherence to sustainable codes.

Furthermore, the “cradle to cradle” concept seems to be a small obstacle for many students. With 23.1 % of them selecting “undecided” when asked about this concept in statement 25, it is evident that there is a knowledge gap in this area. The “cradle to cradle” concept, which refers to a regenerative approach to design, is a critical element of sustainability. The lack of a solid understanding or stance on it could impact the quality and depth of sustainable design practices students can employ.

It is also worth noting the responses to statement 24. This item gauges whether students can justify the added cost of sustainable features to clients. The high percentage of undecided responses suggests that students may not yet feel confident or equipped to communicate the value and benefits of sustainability in a way that justifies higher costs.

Looking at the table, the standard deviations offer valuable insights into the diversity of opinions among the participants. A smaller standard deviation suggests a consensus among the students, whereas a larger one reveals a wider range of opinions.

In summary, this analysis of Table 4 shines a light on the complexities of how these interior design students perceive sustainability. The results reveal an encouragingly high level of support for sustainable design practices. However, they also expose uncertainty and potential gaps in the students' understanding of sustainability, particularly concerning green organizations and certain sustainability concepts.

These findings highlight the importance of addressing these knowledge gaps through enhanced education and discussion within the interior design curriculum. By doing so, we can foster a more comprehensive understanding of sustainability and prepare students to apply these principles more effectively and confidently in their work.

### 8.2 Overall NEP score

An overall NEP score can be calculated to get a general idea of the overall attitude of the participant toward sustainability (see Table 5). According to Ref. [18], an overall NEP score of three is the borderline between a Pro-sustainability attitude and an anthropocentric worldview. In this study, the overall mean score stands at 3.80 (as seen in Table 5), a value generally indicative of a pro-sustainability mindset. However, this aggregate figure does not offer explicit or precise details; it merely supplies a broad impression of the data's direction.

**Table 5:** NEP overall score.

	N	Mean	Std. Deviation
NEP Score Mean	160	**3.8070**	.39116
Valid N (listwise)	160		

Table 5 encapsulates the study participants' New Ecological Paradigm (NEP) scores. The NEP is a widely recognized tool for evaluating individuals' environmental attitudes and beliefs, allowing us to discern whether these leanings veer more towards a pro-sustainability stance.

As depicted in the table, 160 participants filled out the survey (Valid N), with an average NEP score of 3.8070 (Mean) and a standard deviation of .39116 [18]. posits a mean NEP score of three as the threshold distinguishing sustainability attitude. Thus, the study's mean score of 3.8070 suggests that the participants, on average, displayed a pro-sustainability tendency.

A smaller standard deviation would signify homogeneity in the participants' attitudes. Here, a standard deviation of .39116 implies a certain degree of concurrence among the participants regarding their sustainability attitudes.

However, it is crucial to recognize that while the aggregate NEP score offers a useful indicator of the general sustainability orientation among the participants, it fails to shed light on specific facets or dimensions of their attitudes. An in-depth analysis of individual NEP items would be requisite to garner a more nuanced grasp of the participants' sustainability attitudes.

**Fig. 4:**
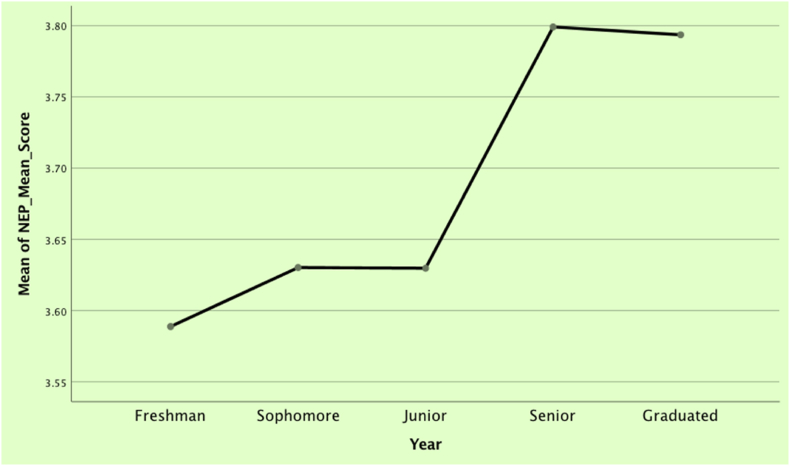
Comparing NEP mean scores based on student level.

Table 6 and Fig. 4 present data from a one-way Analysis of Variance (ANOVA) which compares the mean NEP scores across different student levels in the program. This statistical examination probes the hypothesis that varying student levels could have dissimilar mean NEP scores.

**Table 6:** Comparing NEP mean scores based on student level.

ANOVA					
	Sum of Squares	df	Mean Square	F	Sig.
Between Groups	1.199	**4**	.300	**2.195**	**.072**
Within Groups	21.159	**155**	.137		
Total	22.357	159

From this analysis, we find no statistically significant difference in the NEP scores based on students' levels in the program (F (4,155) = 2.195, p = .072). A p-value exceeding .05 implies that the observed disparities among group means could have emerged by chance, providing insufficient evidence to suggest significant variation in mean NEP scores across diverse student levels.

Despite the absence of statistical significance, a perceptible shift in the mean NEP score for junior-level students is evident, as illustrated in the mean plot. Furthermore, a divide is discerned in the overall mean NEP score between junior and senior students.

This observed variation could be attributed to students' exposure to sustainable design principles throughout their academic progression. It implies that students' attitudes towards sustainability could evolve, accompanied by an enhanced understanding and valuation of sustainable design principles, as they advance in their program. This emphasizes integrating sustainability concepts into the curriculum to shape students' attitudes toward sustainable design practices.

While these findings did not achieve statistical significance, they underscore the opportunity for more rigorous, longitudinal studies to investigate how academic exposure to sustainability concepts influences students' sustainability attitudes over time (Fig. 4).

## 9 Discussion and conclusions

This study examined the attitudes toward sustainability among interior design students at various academic levels. Overall, the findings revealed that participants exhibited a positive outlook toward sustainability and acknowledged its importance for the future of our planet. Most significantly, the students showed a keen interest in the resources and materials sector of the survey, advocating for strategies that minimize waste and promote recycling.

However, a noteworthy response trend was the participants' belief in their entitlement to use natural resources to meet their human needs. While this perspective may initially appear contradictory to sustainable attitudes, it is important to recognize the complementary relationship between resource utilization and sustainability. As asserted by Ref. [21], the understanding that natural resources form the bedrock for human activities is commendable and can be channeled into promoting responsible and sustainable practices in design.

The findings from this study underscore the potential of capitalizing on this recognized understanding as a stepping-stone toward endorsing sustainable design practices. As articulated by Ref. [23], the perception of resource utilization should not be construed as antagonistic to sustainability but rather seen as an impetus to stress the crucial need for responsible stewardship and the incorporation of sustainability within the design process.

This study contributes to the current body of knowledge in the field of interior design education, particularly in Jordan and the Middle East region. The study sheds light on a subject that has not been well explored, focusing on the attitudes of interior design students towards sustainability. The findings offer a comprehensive assessment of the present state of sustainability awareness among interior design students, as well as practical suggestions for enhancing sustainability education in this context.

The study's findings reveal an advanced awareness of sustainability among interior design students. Although they generally hold a positive view, their remarks highlight particular aspects of sustainability that they are interested in and concerned about, such as resource management and recycling. This exemplifies a holistic perspective on sustainability, surpassing mere recognition of its importance and delving deeper into its tangible consequences in the field of design.

Nonetheless, the results also unveiled a clear comprehension gap concerning sustainable practices and strategies amongst numerous participants, thus signaling the necessity for an enhanced sustainability curriculum within design programs. As maintained by Ref. [17], a brief course focusing on sustainability could serve as an instrument to amplify sustainability-oriented attitudes and heighten awareness about ecological issues facing our planet.

This research contributes substantially to our understanding of the sustainability attitudes held by interior design students and how these attitudes are molded. The findings accentuate the necessity of an early induction to sustainability principles and advocate for a comprehensive integration of sustainability throughout educational programs. This strategy could ensure students engage with various aspects of sustainability broadly and informally, thereby fostering a mindset oriented towards sustainability.

By situating the findings within the wider framework of sustainable design education, their importance becomes more evident. By correlating these views with current research, both on a global scale and specifically within Jordan, the study demonstrates their alignment with wider patterns in design education. This contextualization facilitates a deeper understanding of how educational contexts and cultural influences form these perspectives, hence enhancing the findings.

Design educators should expand their pedagogy beyond portraying sustainability as a theoretical concept. They should, instead, embrace it as a process that entails application and implementation, an argument corroborated by Ref. [2]. Moreover, the findings advocate for creating a contemporary course that accentuates sustainability techniques and practices underpinned by evidence-based knowledge. This recommendation becomes particularly salient in light of the misinformation concerning sustainability and climate change that pervades some media channels today.

Because of the positive attitudes towards sustainability manifested by interior design students, it becomes imperative to nurture and build on these predispositions. By offering a comprehensive sustainability education, design programs have the potential to arm students with the necessary skill set to devise sustainable design solutions, thereby positioning them to become catalysts of change in their profession.

The results emphasize the need for design educators to capitalize on students' favorable attitudes towards sustainability. By adopting these mindsets, educators can enhance sustainability education by integrating tangible, experiential activities that reinforce sustainable design concepts. Possible measures could involve overhauling educational programs, incorporating sustainability-oriented initiatives, or providing students with opportunities to engage with sustainable design methodologies in practical contexts.

The study's findings offer valuable insights into the impact of cultural factors on sustainability attitudes within Jordan's cultural context. Design educators may enhance the cultural relevance and effectiveness of sustainability education programs by acknowledging and accommodating these cultural differences. This insight highlights the importance of considering the specific local circumstances while developing sustainability education initiatives, not only in Jordan, but also in other culturally similar environments globally.

When considering the incorporation of ecological sustainability into design education, it is essential to take into account the cultural and educational context, specifically in Jordan. Although sustainability in design education is acknowledged worldwide, the adoption of sustainable techniques in Jordan may encounter distinctive obstacles due to cultural, institutional, and infrastructural shortcomings. A major difficulty is the insufficient focus on sustainability within the Jordanian educational system, potentially leading to a lack of knowledge and experience in sustainable ideas and practices among students. Moreover, variations in cultural perspectives on environmental concerns and sustainability might influence the level of importance placed on incorporating sustainable design principles into the curriculum. Moreover, the incorporation of sustainability into design education may be impeded by institutional constraints such as insufficient resources, obsolete curricula, and a shortage of faculty members with expertise in sustainable design. These challenges can affect students' comprehension and acceptance of sustainable attitudes and practices. By examining the survey findings, it becomes clear that Jordanian interior design students possess a strong interest in sustainability. However, their understanding of specific sustainable techniques is inadequate. This emphasizes the necessity of implementing a thorough and culturally aware strategy for sustainability education in design programs in Jordan. To enhance students' proficiency in sustainable design, Jordanian design colleges should overcome these challenges and incorporate sustainability education into their curriculum. This not only enhances the students' experience but also adds to the overarching objective of fostering ecological sustainability in the field of design.

Lastly, sustaining momentum in research focused on sustainable design education is critical. Future investigations should explore more nuanced aspects of sustainable design, thereby refining the curriculum and equipping students with the skills to tackle the complex sustainability challenges our world currently confronts.

In summary, this research offers an incisive examination of the sustainability attitudes prevalent among interior design students. The results serve as a potent reminder of the imperative to weave sustainability into design curricula, champion responsible resource usage, and recognize the social and cultural factors that shape these attitudes. Through these efforts, we can steer the trajectory of the design industry toward a more sustainable, ecologically cognizant future.

## Recommendations

Based on the findings of the research, the following recommendations are proposed for academic institutions, particularly those providing design programs:

Conduct Continued Research and Assessment: It is important for academic institutions to assess continuously and research students' sustainability attitudes and understanding. Regular assessment will provide feedback on the effectiveness of current pedagogical practices and inform improvements and adjustments in the curriculum. Conversely, continued research can reveal changing trends, insights, and advances in sustainable design that can be incorporated into the teaching material.

Customized Curriculum Enhancement: Develop and improve the curriculum to align with students' advanced comprehension of sustainability. Offer specialized courses that go into certain aspects of sustainability that students have a particular interest in, such as resource allocation and waste management. Integrate interdisciplinary projects that enable students to explore these disciplines through practical, experiential methods.

Foster a Culture of Sustainability: Beyond curriculum changes, institutions should strive to cultivate a culture of sustainability on campus. This could include sustainable practices in campus operations, involvement in sustainability initiatives, hosting sustainability-related events, and promoting sustainability values among staff and students. This approach can reinforce classroom learning and demonstrate the institution's commitment to sustainability.

By adopting the proposed recommendations, academic institutions can greatly improve their students' comprehension and appreciation of sustainable design. As a result, the next generation of designers will be well-prepared with the necessary knowledge and skills to devise sustainable solutions, thereby making a significant impact on improving our world.

Practical Application Emphasize the practical implementation of sustainable design concepts in real-life environments. Promote students' utilization of their knowledge and abilities in sustainable design by offering internships, design projects, and partnerships with industry collaborators. Provide students with the chance to observe the tangible outcomes of sustainable design methods in their localities.

Cultural awareness and sensitivity involve the ability to identify and adapt to cultural variations in attitudes and actions related to sustainability. Develop sustainability education programs that are tailored to the specific cultural needs and sensitive to the local environment. Interact with local communities to gain an understanding of their viewpoints on sustainability, and subsequently integrate those opinions into the curriculum of design education.

These recommendations not only address the specific findings of this study but also contribute to the broader endeavor of integrating sustainability into education. Given the continuously evolving nature of sustainable design, it becomes crucial for academic institutions to remain up-to-date with these advancements, consistently updating their curricula and strategies to cater to the evolving needs of both the industry and society at large. By doing so, these institutions can play a crucial role in fostering a generation of professionals who are well-equipped to tackle sustainability challenges and drive meaningful change in the world.

In conclusion, sustainability in design education is not just a trend or a stand-alone topic; it is an essential aspect of how we should educate future generations of designers. By recognizing this importance and taking actionable steps to integrate sustainability into their programs, universities can better prepare their students for the challenges and opportunities in the viewable design.

## Study limitations and future recommendations

While this research provides valuable insights into the viewpoints of interior design students in Jordan on ecological responsibility and environmental sustainability, it is crucial to recognize certain constraints.

The study's sample consisted of interior design students from a single university in Northern Jordan. While much effort was made to ensure the academic and gender diversity of the sample, it is important to note that the findings may not be generalizable to a broader community of interior design students in Jordan or the Middle East. Subsequent investigations should include including samples from a larger number of colleges or institutions to enhance the representativeness of the sample.

Moreover, it is important to acknowledge that while the survey used in this study was modified from the New Ecological Paradigm (NEP) Scale, it may not have covered the entire range of viewpoints and beliefs related to sustainability in the field of interior design. The NEP Scale is specifically developed to evaluate ecological worldviews and may not fully reflect the complex perspectives that students may have regarding the sustainability of design. Additional inquiry may be necessary to incorporate qualitative approaches or supplemental indicators in order to achieve a more comprehensive understanding of the attitudes and beliefs held by students.

Moreover, the study's cross-sectional approach provides a temporary representation of attitudes. While this method is useful for studying associations between variables, it has limitations when it comes to establishing cause-and-effect links or tracking changes over time. Longitudinal studies can provide a more thorough understanding of how students' attitudes towards sustainability in interior design grow over their academic journey.

Furthermore, the study primarily focused on the attitudes and beliefs as self-reported by the pupils. While self-report measures are commonly used in survey research, they are vulnerable to response bias and may not consistently reflect actual behavior. Additional research in this field could entail integrating impartial measures to evaluate sustainability knowledge and practices in interior design education, such as analyzing coursework assessments or observing real design projects. It is also recommended to have a separate study that compares different cultures students’ attitudes toward sustainability, to understand the cultural roles in this issue.

Despite these limitations, this research provides a useful addition to the growing body of knowledge on sustainability in interior design education. Furthermore, it emphasizes the importance of integrating sustainability ideas into design courses in order to foster environmentally conscious design practices among future interior designers.

## Ethical statement

IRB Approval was obtained from Yarmouk University in Jordan. The Approval number (IRB/2023/420).

## Data availability statement

- Has data associated with your study been deposited into a publicly available repository? No
- Data will be made available on request

## CRediT authorship contribution statement

**Yaman Sokienah:** Writing – review & editing, Writing – original draft, Visualization, Validation, Supervision, Project administration, Methodology, Formal analysis, Conceptualization.

## Declaration of AI and AI-assisted technologies in the writing process

During the preparation of this work the author used Grammarly and Quillbot in order to improve language and readability. After using this tool/service, the author reviewed and edited the content as needed and takes full responsibility for the content of the publication.

## Declaration of competing interest

The authors declare that they have no known competing financial interests or personal relationships that could have appeared to influence the work reported in this paper.
